# Multicentre assessment of transperineal targeted prostate biopsy performed as part of a targeted and systematic biopsy diagnostic strategy in men without previous prostate biopsies

**DOI:** 10.1002/bco2.70020

**Published:** 2025-04-16

**Authors:** Mohammed Sheweita, Liam Blaney, Jon Oxley, Douglas Kopcke, Stefanos Bolomytis, Paul Burn, Adrian Andreou, Jon Heron, Raj Persad, Nick Burns‐Cox, Jonathan Aning, the South West Prostate Dashboard collaborative

**Affiliations:** ^1^ North Bristol NHS Trust Bristol Urological Institute Bristol UK; ^2^ Strategy and Transformation Surrey NHS South, Central and West UK; ^3^ Department of Pathology North Bristol NHS Trust Bristol UK; ^4^ Department of Radiology North Bristol NHS Trust Bristol UK; ^5^ Department of Radiology Somerset NHS Foundation Trust Taunton UK; ^6^ Department of Radiology Royal United Hospitals Bath NHS Foundation Trust Bath UK; ^7^ Population Health Sciences, Bristol Medical School University of Bristol Bristol UK; ^8^ Department of Urology Somerset NHS Foundation Trust Taunton UK

**Keywords:** biopsy concordance, clinically significant prostate cancer detection, systematic prostate biopsy, targeted prostate biopsy, Transperineal biopsy

## Abstract

**Objective:**

To investigate the added value of systematic biopsies in men referred with suspected PCa undergoing visual registration targeted local anaesthetic transperineal prostate biopsies (LATPB) as their first biopsy for MRI‐P visible lesions (MRI Score≥3) in a real‐world setting.

**Patients and methods:**

The outcomes of 2611 biopsy naïve men with MRI Score≥3 who underwent visual registration combined targeted and systematic LATPB at 5 hospitals between 2021 and 2024 were studied. The primary outcome was the clinically significant PCa (csPCa [Gleason≥ 3 + 4 = 7])) cancer detection rate at targeted prostate biopsy without upgrading contributed by the systematic component of the biopsies.

**Results:**

Overall, PCa was diagnosed in 2079/2611 (80%) patients. The targeted biopsy csPCa detection rate in MRI Score 3,4 and 5 lesions was 108/534 (20%), 461/940 (49%) and 865/1137 (76%), respectively. The csPCa detection rate for combined biopsies in MRI Score 3, 4 and 5 lesions was 150/534 (28%), 579/940 (62%) and 959/1137 (84%). The NPV for targeted biopsies for MRI scores 3,4 and 5 lesions were 81.7%, 95% CI = (78.0%, 84.9%), 68.4%, 95% CI = (63.5%, 73.0%) and 55.7%, 95% CI = (48.0%, 63.1%), respectively. Increasing PSA‐D was strongly associated with increased detection of csPCa at targeted prostate biopsy irrespective of MRI score (chi‐square test p < 0.001).

**Conclusions:**

An MRI‐P and targeted prostate biopsy‐only approach should be considered in all biopsy naïve men with MRI score 5 lesions and MRI score 4 lesions with a PSA Density greater than 0.15.

**Patient summary:**

We looked at the difference between sampling a specific area of interest identified by prostate MRI compared to sampling the area of interest and additionally the prostate zones. In our study, we concluded that sampling the area of interest guided by the MRI scan alone can be more beneficial with less risk of missing out on clinically important prostate cancer in real‐life practice.

## 1 INTRODUCTION

The landmark PROMIS study established magnetic resonance imaging of the prostate (MRI‐P) followed by prostate biopsy as the gold standard diagnostic pathway for men with suspected prostate cancer (PCa).^1^ Prospective studies have since demonstrated that MRI‐P followed by targeted prostate biopsy, is non‐inferior to systematic biopsies.^2^, ^3^ Despite this evidence, performing targeted biopsies alone after MRI‐P continues to be considered controversial.^4^, ^5^ Concerns about MRI‐P quality outside of specialist centres, missing information from unsampled prostate areas and that visual registration targeted biopsy can only be performed by experts prevail.^6^, ^7^, ^8^ Contemporary practice has therefore shifted to predominantly performing both targeted and systematic prostate biopsies after MRI‐P in the pursuit of diagnostic certainty.

Outside of clinical trials, targeted biopsy studies have included relatively small sample sizes and heterogenous biopsy techniques with a focus on transrectal biopsy and utilising fusion software.^6^, ^9^, ^10^, ^11^, ^12^ These factors have limited their application to current clinical practice. Furthermore, there is a lack of data describing outcomes from local anaesthetic transperineal prostate biopsy (LATPB) which has been demonstrated to have a superior safety profile and identify more cancer than transrectal prostate biopsy.^13^, ^14^ There is a need for real‐world evidence addressing these deficits, in men referred with suspicion of PCa and to better understand the additional benefit conferred by systematic biopsies when performed as part of a combined targeted and systematic biopsy approach to inform current standards and practice for visual registration LATPB.

The aim of this multicentre study was to analyse the outcomes of combined visual registration targeted and systematic LATPB in a large population of men with an MRI‐P visible lesion undergoing their first prostate biopsy for suspected PCa.

## 2 METHODS

### 2.1 Study population

Biopsy naïve patients with suspected PCa, referred to five hospitals in South West England between January 1st^,^ 2021 and May 9th^,^ 2024, with an MRI‐P visible lesion, undergoing combined visual registration targeted and systematic LATPB were eligible for inclusion.

### 2.2 Study design

The South West Prostate Dashboard (SWPD) was established in 2019 and is a prospectively kept registry of men referred with suspected PCa to hospitals in the South West of England. Data is input into by trained abstractors the SWPD at each institution. The five hospitals contributing to this study had completed regional MRI‐P quality assurance,^15^ had urologists with several years of experience performing LATP biopsies and submitted >92% complete data to the SWPD. We conducted a retrospective analysis of biopsy naïve patients with MRI‐P visible lesions from these institutions in the SWPD database, who had undergone combined LATP biopsy. Over the study period in total 13 376 referrals for men with suspected PCa were received at these institutions (2847 patients at Royal United Hospitals Bath NHS Foundation Trust, Bath, UK, 3139 patients at North Bristol NHS Trust, Bristol, UK, 2636 patients at University Hospitals Plymouth NHS Trust, Plymouth, UK, 1831 patients at Somerset NHS Foundation Trust and 2923 patients at Royal Cornwall Hospitals NHS Trust).

#### 2.2.1 Study definitions

Suspected PCa was defined by either an elevated prostate‐specific antigen (PSA) or an abnormal digital rectal examination (DRE) or both. MRI‐P visible lesions were defined as MRI score 3 (equivocal regarding the likelihood of PCa),4 (likely to be PCa) or 5 (highly likely to be PCa). MRI‐P scores 3,4 or 5 was ascertained from the uro‐radiologist‐reported Prostate Imaging‐Reporting and Data System version 2 (PI‐RADS)^16^ or LIKERT^17^ scores. Pragmatically there was consensus amongst uro‐radiologists at participating centres through quality assurance that: (i) where both LIKERT and PI‐RADS were reported, the highest score allocated was used as the MRI score and (ii) if more than one target lesion was present, the ‘index lesion’ with the highest MRI score was used.

The term combined biopsy will be used to define when both visual registrations targeted and systematic LATPB were performed in the same setting. Clinically insignificant prostate cancer (ciPCa) was defined as a biopsy sample with ISUP GG1(Gl 3 + 3 = 6) only. Clinically significant prostate cancer (csPCa) was defined as the presence of at least a single biopsy core indicating ≥ISUP GG2 (Gleason 3 + 4 = 7) PCa.

### 2.3 Standards of reporting

The Standards of Reporting for MRI‐targeted Biopsy Studies (START) were used to describe the study population, the conduct and reporting of the MRI, the conduct of the biopsy and the results.^12^ MRI‐P at the institutions was performed on 1.5 Tesla and 3 Tesla scanners, no endorectal coil was used at any institution and a maximum of two lesions were identified and labelled. Table S1 details a summary of the MRI‐P‐specific information for patients included in this study.

In the study cohort, the standardised approach to performing LATPB involved using transrectal ultrasound to guide taking targeted biopsies first followed by systematic prostate biopsies. The person performing the biopsy had access to the MRI‐P images and was aware of the location of the lesion on MRI‐P. The biopsy technique and data collection were standardised according to the Ginsburg consensus.^18^ All biopsies were graded with a Gleason score according to the International Society of Urological Pathology (ISUP) 2005 recommendations by at least one specialist uropathologist.^19^

### 2.4 Outcome measures and statistical analysis

A descriptive analysis of the biopsy results from men undergoing combined targeted and systematic LATPB was performed. Targeted prostate biopsy outcomes were compared with those of combined biopsy within the same patient. Regression modelling of the number of targeted and systematic cores taken at LATPB was performed. The primary outcome was the cancer detection rate of patients with an MRI‐P visible lesion undergoing combined prostate biopsy who received a diagnosis of csPCa determined by the targeted prostate biopsy. Secondary outcomes included the proportion of patients diagnosed with ciPCa, the proportion of patients where the targeted prostate biopsy finding was upgraded by the systematic component of the combined prostate biopsy and the cancer detection rates for MRI Score 3, 4 and 5 lesions. McNemar's test was used to statistically evaluate the contribution of the targeted and systematic biopsies. A planned subgroup analysis was performed of patients aged<80 years with a PSA < 20 ng/ml and an MRI stage <T3b. The negative predictive values (NPVs) (the marker of how accurate a negative targeted biopsy is) and the false negative rate of targeted prostate biopsies (the proportion of those with csPCa which would be missed by using targeted biopsies alone) against the gold standard of combined biopsies were calculated for the different MRI Score groups.

## 3 RESULTS

### 3.1 Study demographic

**FIGURE 1:**
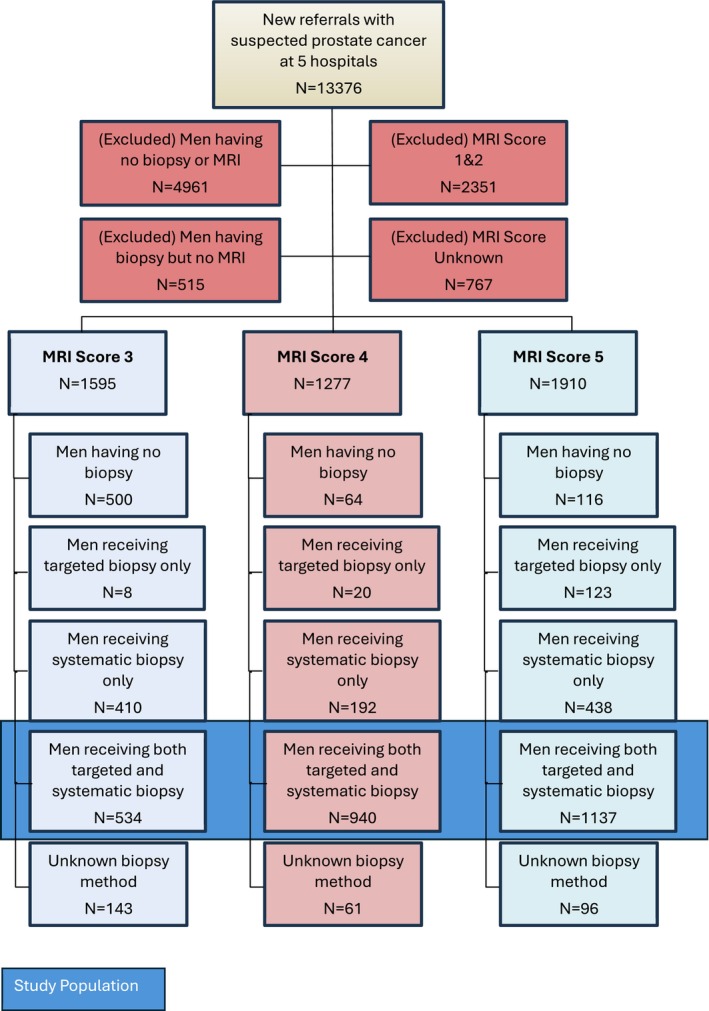
Flowchart of patient cohort included.

The study cohort comprised 2611 patients, 534/1595 (33%), 940/1277 (74%) and 1137/1910 (60%) underwent combined biopsies for MRI Score 3, 4 and 5 lesions, respectively. The flow chart for all patients referred with suspected prostate cancer is illustrated in Figure 1. Overall, 680/4782 (14%) with visible MRI‐P lesions did not undergo a prostate biopsy, the reasons for this are summarised in Table S1. Table 1 shows the demographic and clinical characteristics of all included patients stratified by MRI scores 3, 4 and 5. Table S2 describes the MRI‐P information for the study cohort. The median (IQR) for the number of targeted and systematic prostate biopsies performed per patient in the whole cohort was 3 (IQR 3–5) and 12 (IQR 10–14), respectively. We found that there was no statistical evidence of a difference between the number of targeted prostate biopsies taken at any of the MRI scores (p = 0.687), however, we found strong evidence for differences (p < 0.0001) in the number of systematic prostate biopsies taken. On further investigation, we found that there was no difference between the number of systematic prostate biopsies taken at MRI Score 3 or 4 (mean difference = 0.04 cores (95% CI = [−0.37, 0.44])), however, compared to MRI score group 3, those in MRI Score 5 group had 1 core less statistically taken (11 cores rather than 12 cores) (mean difference = 1.05 cores (95% CI = [0.66, 1.44])) We do not believe that this finding with respect to systematic biopsies to be clinically significant or relevant, merely a reflection of the very large samples used in these analyses.

**TABLE 1:** Patient demographic by MRI score for patients receiving both targeted and systematic biopsy (n = 2611).

	MRI score 3 (n = 534)	MRI score 4 (n = 940)	MRI score 5(n = 1137)
	(median, IQR)	(median, IQR)	(median, IQR)
**Age (years)**	**65, 59–70**	**67, 62–72**	**69, 64–74**
**PSA (ng/mL)**	**6.84, 4.9–9.8**	**7.6, 5.8–10.7**	**10.7, 7.25–17.6**
**MRI prostate volume (mL)**	**52, 36–70**	**43, 32–58.9**	**40, 31–55**
**PSA density (ng/mL2)**	**0.13, 0.09–0.19**	**0.17, 0.12–0.25**	**0.26, 0.17–0.45**
**Index lesion diameter (mm)**	**10, 7–13**	**12, 9–14**	**18, 15–25**
**Number of systematic prostate biopsies taken**	**12, 10–15**	**12, 10–14**	**12, 9–12**
**Number of targeted prostate biopsies taken**	**4, 3–5**	**4, 3–5**	**3, 3–5**
**Number of systematic cores involved with tumour**	**3, 2–5**	**3, 2–5**	**5, 3–7**
**Number of targeted cores involved with tumour**	**2, 1–3**	**3, 2–4**	**3, 2–4**
			
**Clinical T stage**	**(number, %)**	**(number, %)**	**(number, %)**
**2**	**482, 89.9%**	**656, 69.8%**	**335, 29.5%**
**3a**	**7, 1.3%**	**140, 14.9%**	**540, 47.5%**
**3b**	**1, 0.2%**	**12, 1.3%**	**175, 15.4%**
**4**	**0, 0%**	**2, 0.2%**	**17, 1.5%**
**Missing data**	**44, 8.2%**	**130, 13.8%**	**70, 6.2%**
**Clinical N stage**	**(number, %)**	**(number, %)**	**(number, %)**
**0**	**109, 20.4%**	**806, 85.7%**	**1014, 89.2%**
**1**	**1, 0.2%**	**9, 1%**	**51, 4.5%**
**2**	**0, 0%**	**0, 0%**	**1, 0.1%**
**Missing data**	**424, 79.4%**	**124, 13.2%**	**71, 6.2%**

### 3.2 Cancer detection rates of targeted biopsy relative to disease prevalence identified by combined biopsies

Overall, PCa was diagnosed in 2079/2611 (80%) patients undergoing combined LATPB for MRI‐P visible lesions. The prevalence of PCa identified by combined LATPB for MRI Score 3, 4 and 5 lesions was 268/534 (50%), 761/940 (81%) and 1050/1137 (92%), respectively.

**FIGURE 2:**
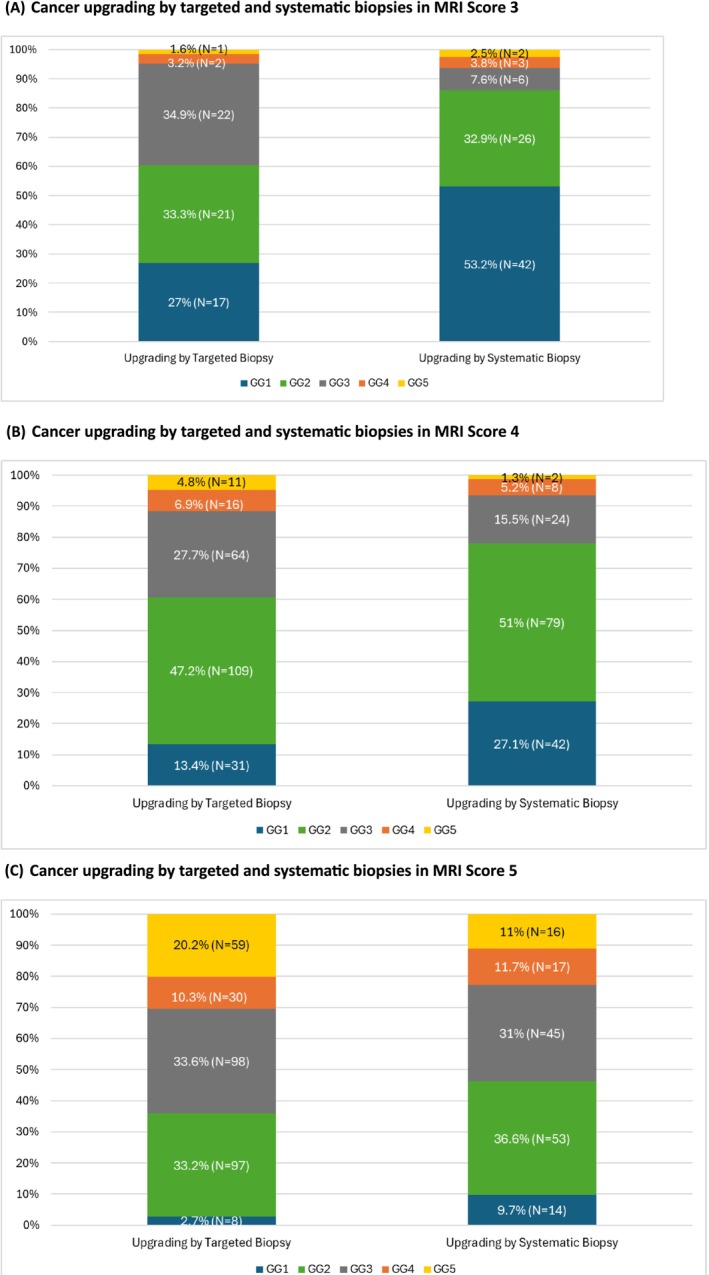
Cancer upgrading by targeted and systematic biopsies. a) Cancer upgrading by targeted and systematic biopsies in MRI score 3. b) Cancer upgrading by targeted and systematic biopsies in MRI score 4. c) Cancer upgrading by targeted and systematic biopsies in MRI score 5.

csPCa was detected by targeted biopsy in 1434/2611 (55%) and 1340/2611 (51%) by systematic biopsy in the same setting when taken as separate entities of the combined biopsy. Tables 2a, b and c illustrate the highest targeted and systematic biopsy ISUP GG outcomes per patient. Figures 2a, b and c illustrate cancer upgrading by targeted and systematic approaches. The csPCa detection rate for targeted biopsy for MRI Score 3,4 and 5 lesions was 108/534 (20%), 461/940 (49%) and 865/1137 (76%), respectively, compared to 150/534 (28%), 579/940 (62%) and 959/1137 (84%) for combined biopsies.

**TABLE 2:** Cross tabulation of highest International Society of Urological Pathology Grade Group (ISUP GG) detected by prostate biopsy method by MRI score.

a. MRI score 3 (n = 534).									
No. of patients in group with targeted bx									
**No. of patients in group with Systematic bx**		**No Cancer**	**GG1**	**GG2**	**GG3**	**GG4**	**GG5**	**Unknown**	**Total**
	**No Cancer**	**254**	**17**	**13**	**5**	**1**	**0**	**0**	**290**
	**GG1**	**42**	**47**	**8**	**7**	**0**	**1**	**11**	**116**
	**GG2**	**9**	**17**	**48**	**10**	**1**	**0**	**6**	**91**
	**GG3**	**5**	**1**	**0**	**8**	**0**	**0**	**1**	**15**
	**GG4**	**1**	**0**	**1**	**1**	**2**	**0**	**1**	**6**
	**GG5**	**0**	**1**	**1**	**0**	**0**	**1**	**0**	**3**
	**Unknown**	**0**	**1**	**0**	**0**	**0**	**0**	**12**	**13**
	**Total**	**311**	**84**	**71**	**31**	**4**	**2**	**31**	**534**
Upgrading by systematic biopsy 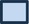									
Both methods identified the same ISUP GG 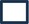									
Upgrading by targeted biopsy 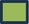									

b. MRI score 4 (n = 940).									
No. of patients in group with targeted bx									
**No. of patients in group with Systematic bx**		**No Cancer**	**GG1**	**GG2**	**GG3**	**GG4**	**GG5**	**Unknown**	**Total**
	**No Cancer**	**154**	**31**	**41**	**17**	**1**	**2**	**2**	**248**
	**GG1**	**42**	**73**	**68**	**16**	**1**	**0**	**32**	**232**
	**GG2**	**18**	**61**	**174**	**31**	**3**	**4**	**21**	**312**
	**GG3**	**9**	**3**	**12**	**43**	**11**	**4**	**2**	**84**
	**GG4**	**1**	**1**	**4**	**2**	**6**	**1**	**0**	**15**
	**GG5**	**1**	**0**	**0**	**0**	**1**	**14**	**1**	**17**
	**Unknown**	**0**	**4**	**4**	**1**	**0**	**0**	**23**	**32**
	**Total**	**225**	**173**	**303**	**110**	**23**	**25**	**81**	**940**
Upgrading by systematic biopsy 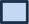									
Both methods identified the same ISUP GG 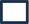									
Upgrading by targeted biopsy 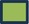									

c. MRI score 5 (n = 1137).									
No. of patients in group with targeted bx									
**No. of patients in group with Systematic bx**		**No Cancer**	**GG1**	**GG2**	**GG3**	**GG4**	**GG5**	**Unknown**	**Total**
	**No Cancer**	**49**	**8**	**31**	**21**	**3**	**10**	**1**	**123**
	**GG1**	**14**	**57**	**66**	**15**	**3**	**9**	**9**	**173**
	**GG2**	**17**	**36**	**245**	**62**	**5**	**15**	**17**	**397**
	**GG3**	**5**	**7**	**33**	**144**	**19**	**15**	**6**	**229**
	**GG4**	**3**	**1**	**5**	**8**	**47**	**10**	**0**	**74**
	**GG5**	**0**	**1**	**3**	**9**	**3**	**80**	**1**	**97**
	**Unknown**	**0**	**3**	**1**	**2**	**0**	**1**	**37**	**44**
	**Total**	**88**	**113**	**384**	**261**	**80**	**140**	**71**	**1137**
Upgrading by systematic biopsy 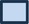									
Both methods identified the same ISUP GG 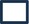									
Upgrading by targeted biopsy 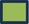									

Table 3 shows an analysis of csPCa (≥ISUP GG2) detection by PSA density (PSA‐D) for men with MRI‐P visible lesions according to targeted prostate biopsy alone. Increasing PSA‐D was strongly associated with increased detection of csPCa at targeted prostate biopsy irrespective of MRI score (chi‐square test p < 0.001). Men with MRI score 5 lesions had greater than 50% prevalence of csPCa irrespective of PSA‐D.

**TABLE 3:** PSA density relationship with MRI score in men where clinically significant (>ISUP GG2) prostate cancer detection.

	Clinically significant prevalence in combined biopsy relative to MRI score	Clinically significant prevalence in targeted biopsy relative to MRI score	Clinically significant prostate cancer prevalence in the PSA density risk groups			
	ISUP≥GG2 prevalence N (%)	ISUP≥GG2 prevalence N (%)	Low (PSA density <0.10) N (%)	Intermediate to low (PSA density 0.10–0.15) N (%)	Intermediate to high (PSA density 0.15–0.20) N (%)	High (PSA density ≥0.20) N (%)
**MRI Score 3**	**155/534 (29%)**	**108/534 (20%)**	**14/164 (9%)**	**31/147 (21%)**	**23/100 (23%)**	**40/123 (33%)**
**MRI Score 4**	**593/940 (63%)**	**461/940 (49%)**	**60/162 (37%)**	**86/210 (41%)**	**92/194 (47%)**	**223/374 (60%)**
**MRI Score 5**	**995/1137 (88%)**	**865/1137 (76%)**	**73/111 (66%)**	**90/128 (70%)**	**105/144 (73%)**	**597/754 (79%)**

ciPCa was detected by targeted biopsy in 370/2611 (14%) and 521/2611 (20%) by systematic biopsy in the same setting when taken as separate entities of the combined biopsy. A McNemar's test for paired proportions showed strong statistical evidence of a difference between these detection rates (p < 0.0001). The ciPCa detection rate for targeted biopsy for MRI Score 3,4 and 5 lesions was 84/534 (16%), 173/940 (18%) and 113/1137 (10%), respectively, compared to 118/534 (22%), 182/940 (19%) and 91/1137 (8%) for combined biopsies. For these three paired comparisons, stratified by MRI score, McNemar's p‐values were 0.002, 0.0002 and < 0.0001, respectively.

### 3.3 Clinically relevant cancer upgrading

Targeted prostate biopsies did not identify any PCa in some cases where PCa was picked up by the systematic component of the combined biopsy: this occurred in 57/534 (11%), 71/940 (8%) and 39/1137 (3%) cases in MRI Score 3,4 and 5 lesions, respectively.

Amongst the patients in whom targeted prostate biopsies identified no cancer or GG1 cancer, combined biopsy upgraded this finding to csPCa GG2 or higher in MRI Score 3, 4 and 5 lesions in 34/395 (9%), 94/398 (24%) and 70/201 (35%) cases, respectively.

Amongst patients in whom no cancer, GG1 or GG2 was identified in targeted prostate biopsies, the combined biopsy identified GG3 or higher in 10/466 (2%), 31/701 (4%), 58/585 (10%) in MRI Score 3, 4, 5 lesions, respectively.

Negative predictive value (NPV) and false negative rate of targeted prostate biopsies compared to combined biopsies for csPCa (≥ISUP GG2).

The NPV for targeted biopsies for MRI scores 3, 4 and 5 lesions were 81.7%, 95% CI = (78.0%, 84.9%), 68.4%, 95% CI = (63.5%, 73.0%) and 55.7%, 95% CI = (48.0%, 63.1%), respectively. The targeted prostate biopsy NPV relationship with PSA Density and MRI Score for clinically significant cancer is described in Table S3.

The false negative rate for targeted biopsies for MRI Score 3,4 and 5 lesions were 23.0%, 95%CI = (17.9%, 28.3%), 10.2%, 95% CI = (8.1%, 12.7%) and 70/931 = 7.5%, 95% CI = (5.9%, 9.4%), respectively.

### 3.4 Subgroup analyses of patients aged <80 years, with MRI stage <T3b N0, PSA ≤ 20

Of the cohort, 2116/2611 (81%) met the subgroup criteria. csPCa was detected by targeted biopsy in 1003/2116 (47%) and 995/2116 (47%) by systematic biopsy in the same setting when taken as separate entities of the combined biopsy. ciPCa was detected by targeted biopsy in 323/2116 (15%) and 461/2116 (22%) by systematic biopsy in the same setting. Cancer upgrading rates and PSA‐D trends mirrored the overall population findings (see Table S4 and S5).

## 4 DISCUSSION

This study aimed to investigate the outcomes of patients with MRI‐P visible lesions undergoing visual registration targeted and systematic LATPB in the same setting as their first biopsy. To the best of our knowledge, this is the largest real‐world multicentre study of a contemporary population of men referred with suspected PCa. Our findings confirm that the practice of combined biopsy is commonplace, accounting for 55% of LATPB performed for MRI‐P visible lesions in our region. In our cohort, the targeted component of combined LATPB identified less ciPCa than the systematic component. Uniquely through the NPV and False negative rate, we have been able to translate the clinical risk and facilitate risk‐based counselling for patients pursuing a targeted‐only biopsy approach. In MRI score 3 lesions a negative targeted biopsy result is unlikely to have missed clinically significant prostate cancer, NPV = 91.4%. Our PSA‐D data potentially enable us to infer additional information to risk stratify discussions about re‐biopsy after a targeted biopsy‐only approach in this situation based on increasing PSA‐D. The false negative rate clearly demonstrates that if a targeted biopsy‐only strategy was employed in our cohort, with increasing MRI score the chances of missing csPCa were reduced, in MRI score 5 this risk was less than 10%.

In randomised clinical trials csPCa (Gl 3 + 4 ≥ 7 or ISUP GG ≥ 2) was detected by targeted prostate biopsy in 36.3% of patients.^20^ In our study cohort overall the targeted prostate biopsy detection rate of csPCa was 55%, and in our subgroup analysis which matched as closely as possible the PRECISION cohort the detection rate for csPCa was 47%. Our data from centres with different multidisciplinary teams, using different MRI machines and visual registration transperineal biopsies demonstrates that targeted biopsy clinical trial findings can be replicated in routine practice. Importantly we have additionally shown in our study what might be missed by adopting a targeted biopsy‐only approach due to the benefit of having a within‐patient control. Even allowing for incorporation bias we have demonstrated limited additional benefit to performing systematic biopsies in all MRI score 5 lesions and MRI score 4 lesions in the presence of a high PSA‐D. Whilst we accept that we have shown a low proportion of csPCa may not be detected if the systematic component of the combined biopsy is not performed, similar to another clinical trial reported recently^21^ we are uncertain as to the lethal potential of such tumours and believe that a period of observation would not lead to the majority suffering harm. Furthermore, we believe that the information provided in our study should provide the basis for shared decision‐making with patients when csPCa is not detected regarding their need for a re‐biopsy, especially in MRI score 3 lesions.

A transperineal prostate biopsy has been demonstrated to have a superior safety profile and demonstrate more cancer than transrectal ultrasound‐guided prostate biopsy^13^, ^14^ making this work timely to reflect on clinical practice moving forwards. The present practice of performing both systematic and targeted prostate biopsies has increased rather than reduced the number of prostate biopsy cores taken. This has had an effect on allied specialities^22^ and the burden will continue to increase with the number of new cases of PCa forecast to rise.^23^ Additionally, the prostate diagnostic pathway contributes a calculable and substantial environmental footprint. There is a need to reduce the number of biopsies and low‐value clinical diagnostic interventions.^24^ We believe our data contributes real‐world evidence to inform the discussion and planning of future strategies. A selective targeted biopsy approach in men with MRI scores 4 and 5 lesions would save procedure time, and pathology time and be more environmentally sustainable.

Our study has several strengths, it represents the largest multicentre consecutive series of men referred with suspected PCa where exclusively only LATP biopsies were employed without fusion system technology. Prospective data collection was performed by independent data inputters reducing the possibility of selection bias. Whilst our study is retrospective, there was broad adherence to a unified biopsy template, as indicated by the median number of systematic and targeted cores obtained. Though we do not know the long‐term outcome of missing the small proportion of csPCa as early detection over lead time bias; we believe that the complete picture provided by knowledge of the combined biopsy results in this study will support shared decision making between patients and clinicians about a targeted biopsy only diagnostic approach based on information and individual risk tolerance. Furthermore, continuous audits utilising platforms such as the SWPD facilitate the maintenance of standards.

The limitations of the present study include that data collection in the SWPD were pragmatic and MRI‐P visible lesions and targeted prostate biopsies were not stratified according to their zonal location precluding an analysis of the impact of ipsilateral versus contralateral systematic biopsies. This data will be captured and investigated in the SWPD in the future. We did not incorporate DRE into our analysis, but this represents the modern practice in the UK of a straight‐to‐test approach without DRE being performed.^25^ Whilst the analysis we performed was retrospective, there was broad adherence to a unified biopsy template within the cohort, as indicated by the median number of systematic and targeted cores which we believe makes our findings robust. We accept though that we do not know the long‐term outcome of missing the small proportion of csPCa as early detection over lead time bias.

In conclusion, in biopsy, naïve men who undergo investigation for suspected prostate cancer and MRI‐P visible lesions are identified a targeted biopsy‐only approach should be considered for all MRI score 5 lesions and MRI score 4 lesions in the presence of a PSA‐D greater than 0.15.

## AUTHOR CONTRIBUTIONS

Liam Blaney, Jon Heron and Jonathan Aning had full access to all the data in the study and took full responsibility for the integrity of the data and the accuracy of the data analysis. **Study concept and design:** Aning. **Acquisition of data:** Liam Blaney, South West Prostate Dashboard collaborative. **Analysis and interpretation of data:** Heron, Aning. **Drafting of the manuscript:** Sheweita, Heron, Aning. **Critical revision of the manuscript for important intellectual content:** Oxley, Kopcke, Bolomytis, Burn, Andreou, Persad, Burns‐Cox. **Obtaining funding:** Persad, Burns‐Cox. **Supervision:** Aning. **Other:** None.

## CONFLICT OF INTEREST STATEMENT

The authors declare that they have no conflict of interest.

## Supporting information


**Table S1** Reasons why patients with visible MRI‐P lesions did not undergo a prostate biopsy
Table S2 MRI‐P information for the study cohort of men undergoing both targeted and systematic biopsy
Table S3 Targeted prostate biopsy NPV relationship with PSA Density and MRI Score in men where clinically significant (>ISUP GG2) prostate cancer detection.
Table S4 Cross tabulation of highest International Society of Urological Pathology Grade Group (ISUP GG) detected by prostate biopsy method by MRI Score in patients aged <80 years, with MRI stage <T3b N0, PSA ≤ 20.
Table S5 Density relationship with MRI Score in men where clinically significant (>ISUP GG2) prostate cancer detection in patients aged <80 years, with MRI stage <T3b N0, PSA ≤ 20.

## Data Availability

Data supporting the results reported in the article can be found in the South West Prostate Dashboard.
