# Supplementary material for: Multicentre assessment of transperineal targeted prostate biopsy performed as part of a targeted and systematic biopsy diagnostic strategy in men without previous prostate biopsies
Source: BJUI Compass. 2025 Apr 16;6(4):e70020. doi: 10.1002/bco2.70020 (PMC12000927; doi:10.1002/bco2.70020)
Supplement: Supplementary file 1 — Table S1 Reasons why patients with visible MRI‐P lesions did not undergo a prostate biopsy Table S2 MRI‐P information for the study cohort of men undergoing both targeted and systematic biopsy Table S3 Targeted prostate biopsy NPV relationship with PSA Density and MRI Score in men where clinically significant (>ISUP GG2) prostate cancer detection. Table S4 Cross tabulation of highest International Society of Urological Pathology Grade Group (ISUP GG) detected by prostate biopsy method by MRI Score in patients aged <80 years, with MRI stage ISUP GG2) prostate cancer detection in patients aged <80 years, with MRI stage <T3b N0, PSA ≤ 20. [file BCO2-6-e70020-s001.docx]

**Supplementary Table 1 Reasons why patients with visible MRI-P lesions did not undergo a prostate biopsy**

| **Reason for no MRI-P / Biopsy** | **Number of patients** | **Median Age, yrs ( IQR)** | **Median PSA, ng/mL ( IQR)** |
| --- | --- | --- | --- |
| **Clinically benign** | **3926** | **75 (67 – 81)** | **7.06 (3.5-11.3)** |
| **Clinically cancer** | **794** | **82 (77 – 86)** | **55.1 (20.65-185.43)** |
| **Patient declined any further investigation** | **68** | **73 (67 - 81.25)** | **13.3 (6.8 – 28)** |
| **Not known** | **173** | **77.5 (72 – 83)** | **13.335 (7.03 - 31.05)** |

**Supplementary Table 2 MRI-P information for study cohort of men undergoing both targeted and systematic biopsy**

|  | **MRI Score 3 (n=534)** | **MRI Score 4 (n=940)** | **MRI Score 5 (n=1137)** |
| --- | --- | --- | --- |
| **Source of MRI Score for cohort** |  |  |  |
| **PIRADS** | **47** | **91** | **105** |
| **LIKERT** | **108** | **294** | **345** |
| **MRI Score PIRADS and LIKERT** | **379** | **555** | **687** |
| **MRI imaging type for cohort** |  |  |  |
| **Multiparametric (MP-MRI)** | **485** | **902** | **1099** |
| **Biparametric (BP-MRI)** | **49** | **37** | **38** |
| **MRI Strength** |  |  |  |
| **1.5 Tesla** | **172** | **382** | **519** |
| **3 Tesla** | **202** | **271** | **293** |
| **Not known** | **160** | **287** | **325** |

**Supplementary Table 3 Targeted prostate biopsy NPV** **relationship with PSA Density and MRI Score in men where clinically significant (>ISUP GG2) prostate cancer detection**

|  | **NPV for clinically significant cancer at targeted biopsy – Percentage ( Number of true negatives / Number of true negatives + Number of false negatives)** | | | |
| --- | --- | --- | --- | --- |
|  | **PSA-D <0.10** | **PSA-D 0.10-0.15** | **PSA-D 0.15-0.20** | **PSA-D ≥0.20** |
| **MRI Score 3** | **88.2% (112/127)** | **82.3% (65/79)** | **74.0% (37/50)** | **72.7% (/55)** |
| **MRI Score 4** | **81.0% (51/63)** | **66.7% (46/69)** | **64.4% (29/45)** | **58.3% (28/48)** |
| **MRI Score 5** | **77.8% (14/18)** | **47.1% (8/17)** | **50.0% (5/10)** | **51.2% (22/43)** |

**Supplementary Table 4 Cross tabulation of highest International Society of Urological Pathology Grade Group (ISUP GG) detected by prostate biopsy method by MRI Score in patients aged <80 years, with MRI stage <T3b N0, PSA≤20**

**Table 4a MRI Score 3 (n=501)**

| **No. of patients in group with targeted bx** | | | | | | | | | |
| --- | --- | --- | --- | --- | --- | --- | --- | --- | --- |
| **No. of patients in group with Systematic bx** |  | **No Cancer** | **GG1** | **GG2** | **GG3** | **GG4** | **GG5** | **Unknown** | **Total** |
|  | **No Cancer** | **237** | **16** | **13** | **5** | **1** | **0** | **0** | **272** |
|  | **GG1** | **41** | **42** | **6** | **5** | **0** | **1** | **11** | **106** |
|  | **GG2** | **9** | **17** | **46** | **9** | **1** | **0** | **6** | **88** |
|  | **GG3** | **5** | **1** | **0** | **7** | **0** | **0** | **1** | **14** |
|  | **GG4** | **1** | **0** | **1** | **1** | **1** | **0** | **1** | **5** |
|  | **GG5** | **0** | **1** | **1** | **0** | **0** | **1** | **0** | **3** |
|  | **Unknown** | **0** | **1** | **0** | **0** | **0** | **0** | **12** | **13** |
|  | **Total** | **293** | **78** | **67** | **27** | **3** | **2** | **31** | **501** |

**Table 4b MRI Score 4 (n=859)**

| **No. of patients in group with targeted bx** | | | | | | | | | |
| --- | --- | --- | --- | --- | --- | --- | --- | --- | --- |
| **No. of patients in group with Systematic bx** |  | **No Cancer** | **GG1** | **GG2** | **GG3** | **GG4** | **GG5** | **Unknown** | **Total** |
|  | **No Cancer** | **143** | **28** | **37** | **16** | **1** | **1** | **1** | **227** |
|  | **GG1** | **40** | **68** | **64** | **15** | **1** | **0** | **30** | **218** |
|  | **GG2** | **15** | **54** | **161** | **29** | **3** | **4** | **19** | **285** |
|  | **GG3** | **8** | **3** | **9** | **39** | **10** | **3** | **2** | **74** |
|  | **GG4** | **1** | **0** | **4** | **2** | **6** | **1** | **0** | **14** |
|  | **GG5** | **1** | **0** | **0** | **0** | **0** | **9** | **1** | **11** |
|  | **Unknown** | **0** | **4** | **4** | **1** | **0** | **0** | **21** | **30** |
|  | **Total** | **208** | **157** | **279** | **102** | **21** | **18** | **74** | **859** |

**Table 4c MRI Score 5 (n=756)**

| **No. of patients in group with targeted bx** | | | | | | | | | |
| --- | --- | --- | --- | --- | --- | --- | --- | --- | --- |
| **No. of patients in group with Systematic bx** |  | **No Cancer** | **GG1** | **GG2** | **GG3** | **GG4** | **GG5** | **Unknown** | **Total** |
|  | **No Cancer** | **40** | **7** | **27** | **10** | **0** | **6** | **0** | **90** |
|  | **GG1** | **13** | **46** | **52** | **12** | **0** | **6** | **8** | **137** |
|  | **GG2** | **15** | **27** | **184** | **47** | **5** | **9** | **12** | **299** |
|  | **GG3** | **3** | **6** | **22** | **89** | **7** | **6** | **5** | **138** |
|  | **GG4** | **2** | **0** | **2** | **5** | **19** | **4** | **0** | **32** |
|  | **GG5** | **0** | **0** | **2** | **2** | **0** | **28** | **0** | **32** |
|  | **Unknown** | **0** | **2** | **1** | **0** | **0** | **0** | **25** | **28** |
|  | **Total** | **73** | **88** | **290** | **165** | **31** | **59** | **50** | **756** |

**Table 5 Density relationship with MRI Score in men where clinically significant (>ISUP GG2) prostate cancer detection in patients aged <80 years, with MRI stage <T3b N0, PSA≤20**

|  | **Clinically significant(≥3+4=7)  prevalence relative to MRI Score** | **Overall Prevalence** | **Clinically significant (≥3+4=7) prostate cancer prevalence in the PSA density risk groups** | | | |
| --- | --- | --- | --- | --- | --- | --- |
|  | **ISUP≥GG2 prevalence** | **ISUP≥GG2 prevalence** | **Low (PSA density <0.10)** | **Intermediate to low ( PSA density 0.10-0.15)** | **Intermediate to high (PSA density 0.15-0.20)** | **High ( PSA density >0.20)** |
|  | **N (%)** | **N (%)** | **N (%)** | **N (%)** | **N (%)** | **N (%)** |
| **MRI Score 3** | **99/501 (20%)** | **145/501 (29%)** | **12/153 (8%)** | **31/145 (21%)** | **22/97 (23%)** | **34/108 (31%)** |
| **MRI Score 4** | **420/859 (49%)** | **536/859 (62%)** | **55/154 (36%)** | **83/204 (41%)** | **90/185 (49%)** | **193/324 (60%)** |
| **MRI Score 5** | **545/756 (72%)** | **638/756 (84%)** | **47/77 (61%)** | **75/107 (70%)** | **92/129 (71%)** | **333/445 (75%)** |
